# Population differences in reproductive resource allocation and heterosis in the invasive vector *Aedes albopictus*

**DOI:** 10.1186/s13071-025-07235-7

**Published:** 2026-01-13

**Authors:** Ayda Khorramnejad, Claudia Alfaro, Stefano Quaranta, Alejandro Nabor Lozada-Chávez, Laila Gasmi, Hugo D. Perdomo, Laurent Roberto Chiarelli, Mariangela Bonizzoni

**Affiliations:** Department of Biology and Biotechnology, University of Pavia, Pavia, PV Italy

**Keywords:** Heterosis, Reproduction, Arboviral vector

## Abstract

**Background:**

An understanding of the traits that favour biological invasions has been considered to be an essential step in predicting which species would become successful invaders. Classical approaches test for differences between invasive versus non-invasive species and emphasize reproduction as a critical phenotype for successful establishment of an invasive species. However, cross-species comparisons underestimate intra-species differences, which may be relevant in species with complex invasion histories.

**Methods:**

We capitalize on the well-characterized invasion history of the arboviral vector *Aedes albopictus*, which has resulted in genetically distinct native, old and invasive populations, and compared the reproductive capacity (fertility and fecundity), development (timing of egg hatching, oviposition patterns and egg hatching) and physiology (blood digestion and nutrient movement during oogenesis) across populations.

**Results:**

The results show that invasive populations are larger in size compared to the *Ae. albopictus* reference Foshan population and have a higher reproductive output than both an old population and the reference Foshan population. The higher reproductive capacity of invasive mosquitoes has both a physiological and genetic basis, and is accompanied by hybrid vigour, albeit at varying degrees across populations.

**Conclusions:**

These findings highlight population-level differences in reproductive traits of *Ae. albopictus* populations that may be associated with their invasion success.

**Graphical Abstract:**

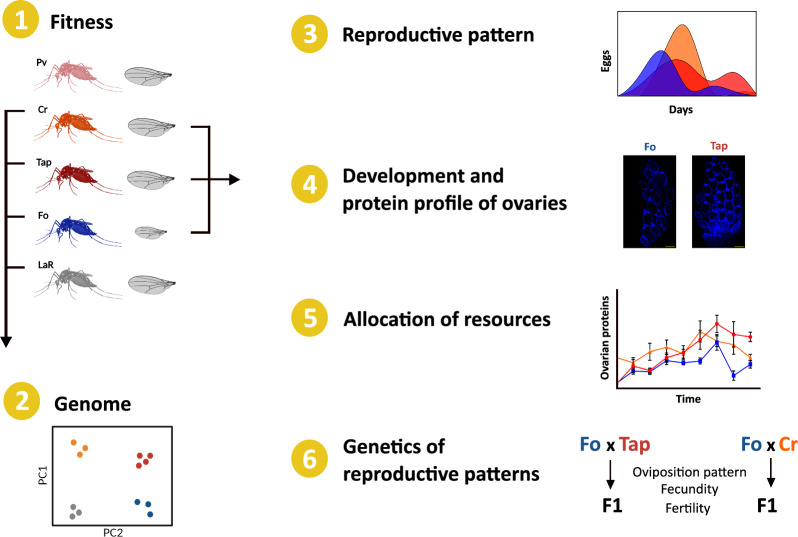

**Supplementary Information:**

The online version contains supplementary material available at 10.1186/s13071-025-07235-7.

## Background

Biological invasions are complex and multifaceted processes that have been recognized since the late 1950 s as a rapidly increasing phenomenon with daunting ecological, sociological and economic consequences [1]. Therefore, understanding the key traits that support a species invasion has been considered to be an important step for the prediction and the management of invasive species [2]. A typical approach is the comparison of species with different invasion success [3–5]. Experimental data obtained from many taxa using a cross-species comparison approach highlight the crucial role of reproduction in the establishment of a species in a new area, following its introduction and preceding further spread [3]. For example, invasive pine species tend to have a small seed mass, which is indicative, in these plants, of a larger seed number, high germinability and higher relative growth rate of seedlings than non-invasive pine species [6]. Data from invasive insects, birds and vertebrate species correspond with life history traits of invasive plants, with successful invasions associated with a high reproductive capacity and short generation time of the invasive species [4, 7–9]. However, traditional approaches that rely on comparisons between invasive and not-invasive organisms tend to neglect intra-species variation, which could be significant in species with highly differentiated populations and/or complex invasion histories.

The Asian tiger mosquito *Aedes albopictus* (Skuse, 1894) is the archetype of a successful invasive species with a complex invasion process. This mosquito, which is native to Asia, colonized islands of the Indian Ocean and the Pacific, such as Hawaii and La Reunion Island, during the spice trade in the seventeenth–eighteenth century [10]. Through the global increases in commerce and human travel during the past century, *Ae. albopictus* moved globally and has currently established populations on every continent, with the exception of Antarctica [11]. Based on this dual wave of colonization, current *Ae. albopictus* populations are called native, old or invasive depending on their origin in Asia, the Indian Ocean and the Pacific or elsewhere in the world [11, 12]. Genetic data from studies performed in the last decades consistently showed that this classification also reflects population genetic differences and that invasive populations often derive from overlaying introductions of mosquitoes of various origins [12–14]. However, it has yet to be empirically tested whether populations with diverse invasion histories differ in their reproductive capacity. The fact that the invasion process of *Ae. albopictus* was mediated by the chaotic distribution of propagules through human activities further suggests genetic mixing among mosquitoes from overlaying introductions. Heterosis and genetic admixture have already been shown to foster the invasion success of multiple plant species, including the highly invasive seep monkeyflower *Mimmulus gattatus* [15, 16] and the common ragweed *Ambrosia artemisiifolia* L [17]. Whether these factors also apply to insect species remains uncertain, with contrasting experimental evidence [18–20].

In the study reported here, we first compared reproductive capacity across geographic populations of *Ae. albopictus* and then used physiological, microscopic and proteomics approaches to investigate mechanisms underpinning observed differences in their reproductive capacity, with a focus on invasive populations. In addition, reciprocal crosses were performed between invasive populations and mosquitoes from the *Ae. albopictus* Foshan (Fo) reference strain to test for heterosis.

## Methods

### Mosquitoes

For this study, we used mosquitoes from the long-established laboratory Fo population [21], together with mosquitoes adapted to the laboratory from eggs collected between 2017 and 2022 from La Reunion Island (LaR), Tapachula, Mexico (Tap), Crema, Italy (Cr) and Pavia, Italy (Pv) [21, 22]. Briefly, eggs were collected from multiple ovitraps across each site or from adults [21, 22]. For the Pv laboratory-adapted population, adult mosquitoes were sampled in the summer of 2022 in sufficient numbers to generate at least 100 adults for the G1 laboratory generation. All five populations were raised in Binder KBWF climatic chambers (Binder GmbH, Tuttlingen, Germany) under constant conditions (28 ± 1 °C; 70–80% relative humidity; photoperiod 12 h:12 h light:dark). The larvae were reared in plastic containers measuring 19 × 19 × 6 cm (BugDorm, MegaView Science Co., Taichung, Taiwan) at a controlled density (approx. 200 larvae in 1 L of water) to prevent competition. Food daily was provided in the form of fish food (Tetra Goldfish Gold Colour; Tetra Werke, Melle, Germany). Adults were kept in 30-cm^3^ cages and fed using cotton soaked with 20% sugar. Adult females were fed commercial defibrinated mutton blood (Biolife Italiana, Monza, Italy) using a Hemotek blood membrane feeding system.

### Fitness assessment

Fitness evaluation was carried out within the first 10 generations after colony establishment for the Tap, Cr, Pv and LaR mosquito populations. Given the differences in the collection time among these populations, fitness assessments were conducted using the same conditions and protocols—but at different times—for each field-derived population, but with each fitness assessment carried out in parallel with fitness assessment of Fo mosquitoes. For each population, three replicates of 100 eggs each were hatched in plastic containers (17 × 6.5 × 12 cm) containing 200 ml of water and reared under the standard conditions described in section Mosquitoes. The larval to pupal developmental time was recorded until pupation. Emerging adults were sexed. In parallel, six more replicates of 100 eggs were hatched for each population in 200 ml of water for further measurements, and samples of 50 females and 50 males was collected for wing length measurement as a proxy for body size, as previously described [22]. For each population, a total of 200 females and 200 males were separated at emergence, and the survival of each individual was monitored until death. Oviposition pattern, fecundity and fertility were also monitored. Briefly, at least fifty 3- to 5-day-old females were placed in cages with 50 males to mate. After 7 days, females were offered a blood meal, following which only fully engorged females were collected, with each individual placed in a separate circular cup. We provided a damp filter paper for egg deposition 2 days after the blood meal and monitored the number of eggs laid by each female for the following 6 days to determine the patterns of oviposition. We also measured the total number of eggs laid per female (fecundity). Each filter paper was then dried for 2 days and subsequently placed in water for egg hatching; the percentage of hatched eggs per female (fertility) was recorded. In parallel, we took a minimum of thirty 3- to 5-day-old mated females per population and weighed them on a microbalance (Mettler AC100; Mettler-Toledo GmbH, Giessen, Germany) before and after the blood meal; the weight difference corresponded to the amount of blood ingested (blood intake). Reproductive fitness assessment for Fo strain mosquitoes showed temporal stability (Additional file 1: Figure S1).

The Prism 9.1 software program (GraphPad Software, San Diego, CA, USA) was used for statistical analyses of life history traits. We verified the normality of each dataset with the Shapiro–Wilk test and proceeded accordingly. Differences in wing length, fecundity and fertility among the different laboratory-adapted populations were tested using one-way analysis of variance (ANOVA), followed by Tukey’s multiple comparison [23]. We compared larval to pupal developmental time, sex ratio, wing size, weight and blood intake using the Student t-test [23] and tested differences in longevity using the Log-rank (Mantel Cox) analysis [23]. Oviposition patterns were compared using a cubic spline analysis [23].

### Reciprocal crosses

We performed reciprocal crosses between mosquitoes from the Tap and Fo populations and from the Cr and Fo populations, respectively, by exposing 20 newly emerged females from one population to seven newly emerged males from the other population for 7 days; we then provided a blood meal and provided a damp filter paper for egg deposition. The F_1_ eggs were hatched and the progenies were interbred. Wing length, fecundity and fertility of the F_1_ mosquitoes were determined as described in section Fitness assessment. Wing length, fecundity and fertility were compared among the progeny of each cross and also with the parental Tap, Cr, and Fo populations, using a Mann–Whitney test in Prism 9.1 software (GraphPad Software) because the data were not parametric.

### Protein and lipid content

A total of thirty-six 3- to 5-day-old female mosquitoes were assessed for protein and lipid content before the blood meal and at 24 and 96 h post blood meal (hpBM). The fat body of each individual mosquito was dissected in 75% ethanol and then weighed using a microbalance (Mettler AC100; Mettler-Toledo GmbH), following which the samples were homogenized individually in 180 μl of lysis buffer (100 mM KH_2_PO_4_ [Sigma-Aldrich, St. Louis, MO, USA], 1 mM DTT, 1 mM EDTA [Thermo Fisher Scientific, Waltham, MA, USA], pH 7.4) with pestles. The samples were stored at − 20 °C until processing to quantify the protein and lipid content using a colorimetric assay as previously described [22]. Following this same procedure, wer determined the protein content of the fat body and ovaries of 216 females that were sampled at 12, 24, 48, 60 and 72 hpBM. We compared protein time and lipid course using two-way ANOVA tests followed by Sidak’s multiple comparison tests using Prism 9.1 (GraphPad Software). Measurements of energy reserves were conducted in parallel for all tested populations.

### Midgut trypsin-like activity

For the measurement of midgut trypsin-like activity, we provided a blood meal to 3- to 5-day-old females and subsequently sampled fully engorged females at 6, 24, 36, 48 and 60 hpBM; a sample of 3- to 5-day-old females (*n* = 144) were also sampled before the blood meal. The midguts of each mosquito were dissected on ice and trypsin-like activity subsequently measured as described previously [24], with slight modifications. Briefly, we homogenized each sample in 100 μl of an extraction buffer (20 mM Tris/20 mM CaCl_2_ [Sigma-Aldrich], pH 8) using a pestle, followed by centrifugation for 2 min at 14000* g*, 4 °C. The supernatants were collected and stored at − 80 °C until measurements were performed. For sugar-fed samples and samples collected 6 hpBM we used aliquots of 10 μl; for samples from all other time points we used aliquots of 5 μl and added these to 100 μl of 4 mM Nα Benzoyl-L- Arginine-p-Nitroanilide (BApNA; Sigma-Aldrich) in wells of a 96-well plate. The plates were incubated at 37 °C for 10 min, followed by an absorbance reading at 405 nm in a CLARIOstar plate reader (BMG Labtech, Ortenberg, Germany). Trypsin-like activity was quantified using a standard absorbance curve generated using 20 μg of bovine serum trypsin (Sigma-Aldrich) with BApNA standards (8.96, 4.48, 2.24, 1.12, 0.56, 0.28 and 0.14 mM).

### Microscopic analysis of mosquito ovaries

A blood meal was provided to 3- to 5-day-old female mosquitoes, and fully engorged females were subsequently samples in groups of five at 24, 30, 36, 42, 48, 72 and 96 hpBM. The ovaries of each female were dissected in 75% ethanol, following which the tissues were fixed in a Canois solution (60% ethanol [Sigma-Aldrich], 30% chloroform [Sigma-Aldrich] and 10% acetic acid [Sigma-Aldrich]) for up to 1 week. The samples were then rinsed 3 times with 1 × phosphate buffered saline (PBS) and permeabilized with permeabilization solution (0.2% Triton [Sigma-Aldrich], 1 × PBS and 1% bovine serum albumin [BSA; Promega, Madison, WI, USA]) for 1 h. As a final step, the ovaries were stained with 4′-, 6-diamidino-2-phenylindole (DAPI [Thermo Fisher Scientific], diluted 1:50 in 1 × PBS) for 5 min before mounting the samples on the slide using polyvinyl alcohol medium (Sigma-Aldrich). Images were taken with a TCS Sp8 confocal microscope (Leica Microsystems, Wetzlar, Germany) at Centro Grandi Strumenti at the University of Pavia (https://cgs.unipv.it/?page_id=84). The complete set of images can be viewed at 10.6084/m9.figshare.30784160.

### Proteomic analyses of ovaries

A blood meal was provided to 3- to 5-day-old female mosquitoes, and either 1 or 5 days later, we sampled 100 fully engorged females to dissect ovaries in 75% ethanol under a dissection stereoscope (Leica ZOOM 2000; Leica Microsystems). Ovaries from females of the same population were pooled and collected at the same time point post blood meal for protein extraction following Geiser et al. [25], with some modifications. Briefly, tissues were homogenized in 500 μl of lysis buffer (7 M urea, 2 M thiourea, 50 mM HEPES pH 8, 75 mM NaCl, 1 mM EDTA and a cocktail of 1 × Halt proteases inhibitors) with a pestle, and then step-wise incubated for 30 min on ice, sonicated using a probe in ice and centrifuged at 12,000 rpm for 10 min at 4 °C. For the quantification of protein content, we collected the supernatant and performed the Bradford assay [26]. Samples were then subjected to sodium dodecyl sulfate-polyacrylamide gel electrophoresis (SDS-PAGE) followed by an in-gel digestion. Briefly, 30 μg of proteins of each sample were loaded onto an SDS-PAGE gel (stacking 5%, resolving 12%) and proteins were separated in an electrophoretic run at 200 V for 40 min. The resulting profile was divided into seven fractions, and the gel was cut accordingly. Each gel piece was destained by covering it with a sufficient volume of a 100 mM ammonium bicarbonate + 50% acetonitrile solution for 15 min. This washing step was repeated until the gel pieces were completely destained. The gel pieces were then dehydrated in pure acetonitrile and incubated at room temperature; the acetonitrile-treated gel pieces were later dried at 60 °C for 5 min. The gel pieces were reduced in a freshly prepared 10 mM dithiothreitol (DTT) solution, with incubation at 37 °C for 30 min, followed by alkylation with freshly prepared 55 mM iodoacetamide (Sigma-Aldrich) and incubation for 45 min at 60 °C. These samples were washed twice with 200 μl of 100 mM ammonium bicarbonate before a second dehydration with 200 μl of pure acetonitrile. We then performed protein digestion by incubating samples on ice for 30 min with 50 μl of 20 ng/μl trypsin in 100 mM ammonium bicarbonate, followed by overnight incubation at 37 °C with sufficient 100 mM ammonium bicarbonate to avoid gel drying. The following day, each gel piece was treated with 100 μ of a 50% acetonitrile/5% formic acid solution for 15 min at 37 °C to extract proteins. The proteins of fragments coming from the same gel fragment were pooled and dried in a Speed Vac® without exceeding 30 °C for 4 h. Peptides were resuspended in 20 μl of distilled water and 1 μl of formic acid and stored at − 20 °C until mass spectrometry analysis.

The proteomic profiles of each sample were obtained via liquid chromatography–mass spectrometry (LC/MS) performed at the Centro Grandi Strumenti of University of Pavia (https://cgs.unipv.it/?page_id=96) using the LC/MS–MS LCQ Fleet instrument (Thermo Fisher Scientific). Mass spectra were generated using SCIEX software (Sciex) and proteins were identified using a threshold of at least one distinct peptide per protein with 95% confidence and a personalized database, which comprises all *Ae. albopictus* proteins (AalbF2 assembly) and *Aedes aegypti* proteins (AaegL5.3 assembly) as obtained from VectorBase (https://vectorbase.org/vectorbase/app).

### Functional gene annotation and enrichment

Gene Ontology (GO) functional assignment and gene enrichment of protein-coding genes were done following Lozada-Chávez et al. [27]. This process allowed the annotation of 69% of the *Ae. albopictus* proteome. Our custom annotation database was used for GO enrichment analysis with clusterProfiler v4.2.2 [28] to identify functional groups that were enriched in proteins identified through the LC/MS analyses of the ovaries. *P*-values (*P* ≤ 0.05) obtained with clusterProfiler were corrected for multiple tests with the Benjamini–Hochberg procedure, and redundant enriched GO terms in each major GO classification were removed with clusterProfiler (‘simplify’ function).

### Genomic analyses

Whole-genome sequencing (WGS) data from pools of 40 mosquitoes/population were used for the genomic analyses. Raw sequencing reads are available under the NIH BioProject number PRJNA1080321. We evaluated the quality of raw sequencing reads with FastQC (v. 0.12.1) [29], and trimmed adapters using fastp (v. 0.23.2) [30]. The resulting reads were aligned with the *Ae. albopictus* genome (AalbF2 assembly, VectorBase rel. 55) [31] using the bwa-mem2 aligner (v. 2.2.1) [32]. The pairwise fixation index (Fst) was calculated across the genome and Tajima’s D genetic diversity (Additional file 2: Tables S1, S2) was calculated using grenedalf (v. 0.3.0) [33] with the PoPoolation methods [34]. Both metrics were computed by excluding nucleotide positions with base counts < 2 (–filter-sample-min-count 2), coverage < 5 (–filter-sample-min-coverage 5), minimum mapping quality < 20 (–sam-min-map-qual 20) and minimum base quality score < 20 (–sam-min-base-qual 20).

## Results

### Invasive mosquitoes are larger and have a higher reproductive output than old and long-laboratory-adapted populations

Laboratory mosquito populations were established from invasive *Ae. albopictus* mosquitoes sampled in Tapachula, Mexico (Tap) and Crema, Italy (Cr) and Pavia, Italy (Pv) [21, 22]. We also adapted eggs collected on the Indian Ocean Island of La Reunion (LaR) to laboratory conditions; this population is referred to here as “old” because it belongs to the first wave of *Ae. albopictus* colonization outside the native range [11, 12]. We measured the body size and the reproductive output (fecundity and fertility) of mosquitoes of these populations within the first 10 generations of laboratory colonization, and compared these parameters to those of mosquitoes of the *Ae. albopictus* reference Foshan (Fo) strain [21].

**Fig. 1:**
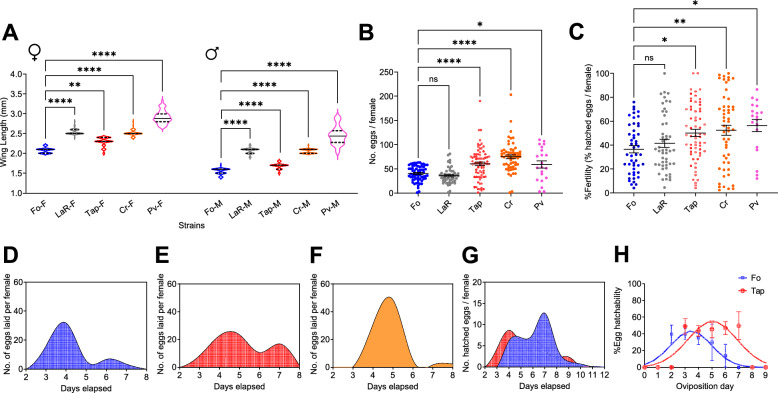
Phenotypic variations among the *Aedes albopictus* populations tested. **A** Wing length of at least 50 females (F) and 50 males (M) of all populations. **B** Number of eggs laid per female per population. **C** Percentage of larvae developed from the eggs deposited by each female.** D–F **Cubic spline curves show the oviposition patterns during the first gonadotrophic cycle (GC) of Fo (**D**), Tap (**E**) and Cr (**F**) mosquitoes. **G** Cumulative pattern of egg hatching during the first GC. **H** Viability of each batch of eggs deposited by Fo and Tap females in relation to their deposition day during the first GC. Error bars represent the 95% confidence intervals. Asterisks indicate significant differences at *P < 0.05, **P < 0.01 and ****P < 0.0001; Ns, not significant. In all panels, data on Fo are in blue, data on Tap are in red and data on Cr are in orange. Fo, *Ae. albopictus* Foshan reference strain; LaR, Tap, Cr, Pv, laboratory mosquito populations established from eggs collected at La Reunion Island, Tapachula (Mexico), Crema (Italy) and Pavia (Italy), respectively

We observed that mosquitoes of both sexes derived from invasive populations, namely the Tap, Cr and Pv populations, have a wider wing length than Fo mosquitoes and a higher reproductive output compared to mosquitoes from both the old population of LaR and Fo mosquitoes, with the reproductive output not differing significantly among invasive mosquitoes (Fig. 1a–c), Additional file 2: Table S3). Using WGS data, we also verified that the tested populations are genetically different, with levels of genetic diversity and pairwise Fst values comparable to those estimated across invasive, old and native *Ae. albopictus* populations, albeit Fo mosquitoes have lower genetic diversity than populations recently adapted to laboratory conditions, as previously noted [12, 21, 35] (Additional file 2: Tables S1, S2).

### The higher reproductive capacity of invasive mosquitoes correlates with a delay in oviposition

Having established that genetically the laboratory mosquito populations we established from invasive *Ae. albopictus* mosquitoes (LaR, Tap, Cr, Pv) and the Fo reference strain reflect the observed distinction among native, old and invasive populations and that the fitness traits of Fo mosquitoes have been maintained constant across generations (Additional file 1: Figure S1), we checked if the higher fecundity of invasive Tap and Cr mosquitoes correlates with a different oviposition pattern with respect to that of Fo mosquitoes. We therefore counted the number of eggs deposited daily from the second to the eighth day pBM. The results revealed delayed oviposition in both Tap and Cr females with respect to Fo females, with peak oviposition occurring in the latter population between 3 and 4 days pBM (Fig. 1d). More than 95% of Fo females deposited their eggs in one or two batches, each with an average (± standard deviation [SD]) clutch size of 20.38 ± 2.99 eggs (Fig. 1d); fewer than 2% of the tested females laid > 2 clutches of eggs throughout the 6 days of our observation period. In contrast, both Tap and Cr mosquitoes had an oviposition peak between 4 and 5 days pBM (Fig. 1e, f), with an average clutch size of 60.05 ± 3.80 eggs for Tap mosquitoes and 74.88 ± 4.05 eggs for Cr mosquitoes. The Tap mosquitoes showed a second peak of oviposition at 7 day pBM; 21.12% ± 9.47% of Tap mosquitoes laid > 2 clutches of eggs, with an average clutch size of 22.80 ± 0.54 eggs (Fig. 1e). Given the presence of two oviposition peaks in Tap and Fo mosquitoes, we tested if egg hatching parallels the oviposition pattern by individually assessing the fertility of females from day 2 to day 8 pBM. We observed that the first batch of eggs deposited by Tap females hatched faster than the first batch produced by Fo females (Fig. 1g). Furthermore, the hatchability of Tap eggs remained constant independently of their oviposition day (Fig. 1h), while that of Fo eggs peaked 2 days after oviposition (38.63% ± 6.43%), then gradually declined. We also observed that while the higher reproductive output of Tap mosquitoes extends from the first to the second GC (Additional file 3: Figure S2), Tap mosquitoes started laying eggs earlier in the second GC, with an oviposition peak at 2 days pBM (Additional file 3: Figure S2).

### The delay in oviposition of invasive females has physiological bases

**Fig. 2:**
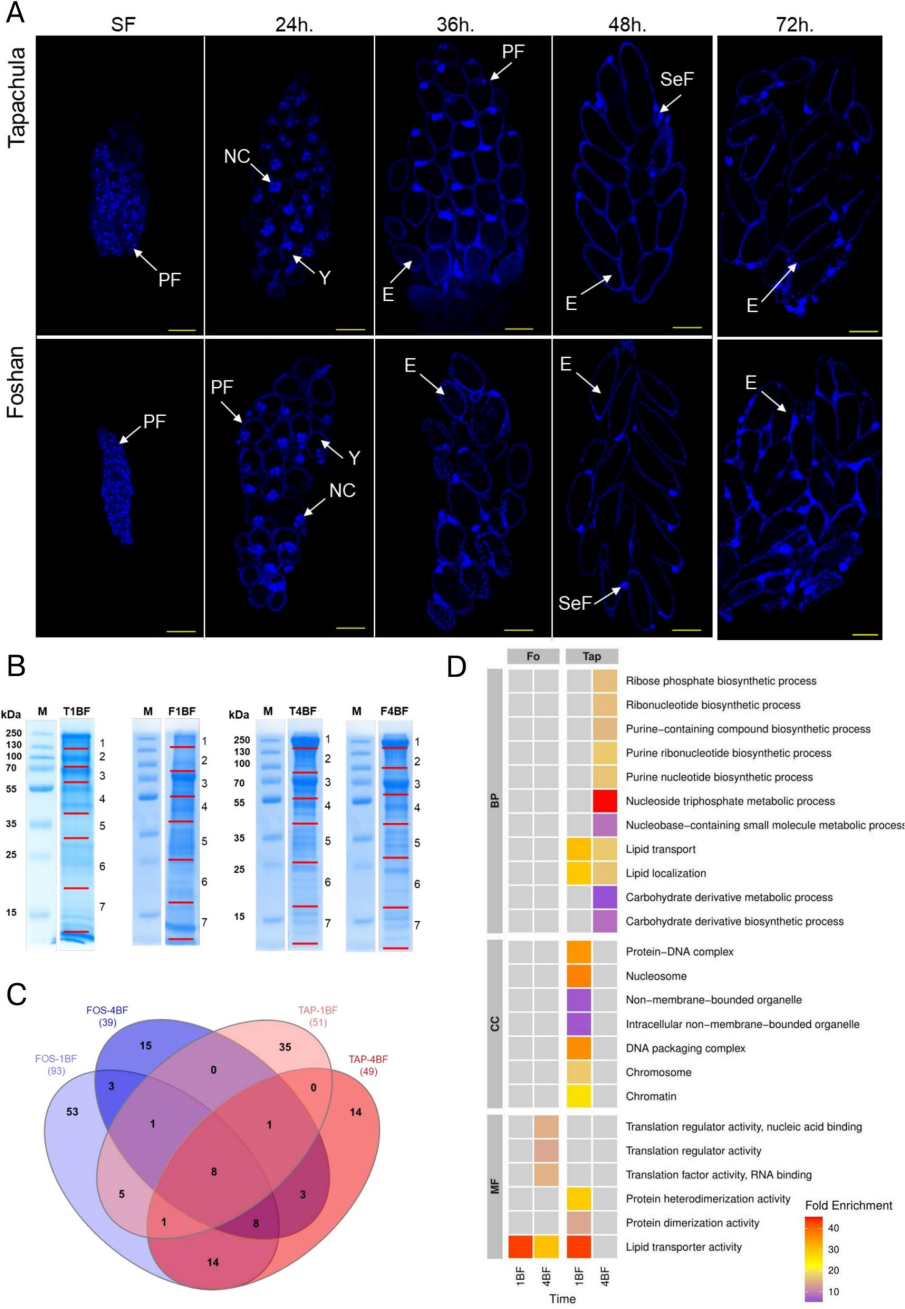
Morphology and proteomics of ovaries. **A** Micrographs of ovaries of female mosquitoes from the Foshan reference strain (Fo) and Tapachula (Tap) population that had been sugar-fed (SF) and subsequently sampled at 24, 36 and 48 hpBM, then stained with DAPI. Arrows point to primary follicles (PF), nurse cells (NC), yolk (Y), eggs (E) and secondary follicles (SeF). Yellow scale line: 50 µm. **B** Sodium dodecyl sulfate-polyacrylamide gel electrophoresis (SDS-PAGE) analysis of ovarian proteins extracted from of ovaries of female mosquitoes of the Tap (T) and Fo (F) populations, dissected one (1) and four (4) days post blood meal (BF). The M lanes correspond to the PAGERuler Plus Prestained Protein Ladder (Thermo Fisher Scientific). Red lines mark the gel slices that were excised. **C** Number of proteins identified in each condition/population. **D** Functional enrichment of proteins from ovaries of Fo and Tap mosquitoes sampled 1 and 4 days pBF. BP, Biological processes; CC, cellular constituents; MF, molecular functions

The observed delay in the oviposition pattern of invasive versus Fo females could be a behavioural trait by which mosquitoes retain mature eggs in their ovaries, or it could result from the physiology of egg production. To discriminate between these alternatives, we combined two approaches, focusing on Tap and Fo mosquitoes based on their two-peak oviposition pattern. First, we studied egg development in maturing ovaries by acquiring micrographs at intervals between 6 and 72 hpBM, starting at 24 hpBM (Fig. 2a). Second, we studied temporal changes in ovarian proteins (Fig. 2b–d).

Images of the ovaries of Fo and Tap females at 24, 30, 36, 48 and 72 hpBM were captured and analyzed in comparison to the images of ovaries of sugar-fed (SF) females (Fig. 2a). Primary follicles with little or no yolk were observed in all SF females (Fig. 2a). Primary follicles with nurse cells and an increasing amount of yolk in the ovaries were observed in both Fo and Tap females sampled at 24 and 30 hpBM; at 36 hpBM, we observed a clear difference in the images of Fo and Tap mosquitoes (Fig. 2a): mature eggs and few primary follicles were observed in the ovaries of Fo females, whereas ovarioles containing mostly primary follicles with high amounts of yolk were seen in Tap females. Mature eggs were seen in the ovaries of both populations after 48 hpBM. These results support the conclusion that the observed delay in the oviposition of Tap with respect to Fo females is caused by different dynamics of egg formation and not by egg retention in Tap mosquitoes.

We subsequently compared the protein profile of ovaries of fully-engorged blood-fed Tap and Fo mosquitoes at 1 and 4 days pBM; this time interval represents the trophic and posttrophic stages of vitellogenesis in mosquitoes, respectively [36, 37]. The quantity of total proteins extracted from the ovaries at 1 day pBM did not differ between Fo and Tap mosquitoes (mean ± SD: 47.96 ± 4.99 µg/mg in Fo and 42.08 ± 6.93 µg/mg in Tap mosquitoes), but the complexity and abundance of proteins did differ (Fig. 2b). At 4 days pBM, protein quantity was higher in the ovaries of Tap mosquitoes than in those of Fo mosquitoes (the total mean protein content was 107.72 ± 17.65 µg/mg in Fo and 163.92 ± 18.88 µg/mg in Tap; *t* = 2.919,* df* = 44, *P-*value = 0.0110; Additional file 4: Figure S3), while the SDS-PAGE profile of total ovarian proteins appeared visually similar. The Tap ovaries sampled 1 day pBM were enriched in proteins associated with carbohydrate metabolic process and proteins related to non-membrane-bounded organelles, which are associated with cell structure and motility [38] (Fig. 2c, d; Table 1; Additional file 2: Table S4). At the same time point pBM, proteins found uniquely in Fo ovaries were enriched in lipid transporter activity and proteins associated with the development of oocytes and the eggs. A total of 15 proteins (Fig. 2c; Table 1), among which the presence of vitellogenin precursor (AALF008766) is notable (Additional file 2: Table S4), were found in the ovaries of both Fo and Tap females 1 day pBM (Fig. 2c, d). At 4 days pBM, we detected proteins in Tap ovaries that were enriched in functions such as carbohydrate metabolic processes, proteolysis and lipid localization related to oocyte development [39]. At the same time point, proteins exclusive to ovaries from Fo female mosquitoes were enriched in proteins linked to translation regulatory activity, lipid transporter and proteins that mediate the late stages of oogenesis and egg activation [40]. A total of 20 proteins, some of which are involved in oogenesis (e.g. vitellogenin precursor [AALF008766]) were found in the ovaries of Fo and Tap mosquitoes at 4 days pBM (Table 1). Based on the observed delay in oviposition in Tap mosquitoes with respect to their Fo counterparts, we compared the proteins detected in the ovaries sampled at 4 days pBM in Tap and 1 day pBM in Fo females (Fig. 2c; Table 1). We found 31 proteins associated with oogenesis, carbohydrate metabolic process, protein biosynthesis and glycolytic process. Importantly, none of these 31 proteins was found in the ovaries of Tap mosquitoes sampled at 1 day pBM, except for vitellogenin-A1 precursors (AALF008766 and AAEL010434) (Additional file 2: Table S4). Collectively, proteomics data support a delay in the biochemical activity of Tap mosquitoes for oogenesis in alignment with fitness results and data from ovarian micrographs.

**Table 1:** List of the proteins identified in the ovaries of female mosquitoes of the Foshan reference strain and Tapachula population at 1 and 4 days post blood meal, and their gene ontology based on either molecular function or biological process

Fo			Tap		
Accession no.	Name	GO (MF/BP)	Accession no.	Name	GO (MF/BP)
One day pBM					
AALF000045	Unspecified product	Protein catabolic process	AALF000221	Odorant receptor	Sensory perception of smell
AALF002182	Alpha-amylase	Carbohydrate metabolic process	AALF000790	Histone H2B	Nucleosome assembly
AALF002986	AMP dependent ligase	Catalytic activity	AALF000911	Unspecified product	Nucleic acid binding
AALF003502	Kynurenine aminotransferase	Kynurenine metabolic process	AALF001196	C-Type Lectin (CTL)-mannose binding	Carbohydrate binding
AALF004064	Unspecified product	Catalytic activity	AALF002028	Ran-binding protein	Intracellular transport
AALF007667^a,b^	Elongation factor 1-alpha	Translational elongation	AALF002835	Histone H2A	Nucleosome assembly
AALF007724	ATP synthase subunit beta	Proton motive force-driven ATP synthesis	AALF005503	Histone H4	DNA-templated transcription initiation
AALF008379^a,b^	Unspecified product	Lipid transport	AALF007667^a^	Elongation factor 1-alpha	Translational elongation
AALF008766^a,b^	Vitellogenin-A1 precursor	Lipid transport	AALF007713	Profilin	Larval central nervous system remodeling
AALF009209^a,b^	Serine/threonine kinase	Protein phosphorylation	AALF007792	Unspecified product	Protein binding
AALF010133^a,b^	Elongation factor 1-alpha	Translational elongation	AALF008379	Unspecified product	Lipid transport
AALF010343^b^	Unspecified product	Carbohydrate binding	AALF008382	Serine-type enodpeptidase 2C	Proteolysis
AALF011822	Leucine-rich immune protein	Protein binding	AALF008646	Histone H2B	DNA binding
AALF011930	Unspecified product	Nucleic acid binding	AALF008766^a^	Vitellogenin-A1 precursor	Lipid transport
AALF012772	Tyrosine-protein kinase receptor	Protein phosphorylation	AALF008869	Unspecified product	mRNA processing
AALF013670	Unspecified product	Signal peptide processing	AALF008985	Histone H2B	Nucleosome assembly
AALF014888	Unspecified product	Iron-sulfur cluster assembly	AALF009114	Histone H2B	Nucleosome assembly
AALF015638	Shc transforming protein	Intracellular signal transduction	AALF009200	Unspecified product	Transferase activity
AALF016323	Unspecified product	MAPK cascade	AALF009209^a^	Serine/threonine kinase	Protein phosphorylation
AALF017232^b^	Cathepsin b	Proteolysis	AALF010133^a^	Elongation factor 1-alpha	Translational elongation
AALF017233^b^	Unspecified product	Proteolysis	AALF010136	Histone H2A	Nucleosome assembly
AALF018275^b^	Unspecified product	Proteolysis	AALF010978	Odorant receptor	Sensory perception of smell
AALF018287	Histone H2A	Chromatin organization	AALF012625	Unspecified product	Nucleic acid binding
AALF018448	Unspecified product	Lyase activity	AALF013617	Histone H2A	Somatic stem cell population maintenance
AALF018477^a^	Serine/threonine protein kinase	Protein phosphorylation	AALF015445	Nephrin	NA
AALF019930^a,b^	Unspecified product	Lipid transport	AALF018477^a^	Serine/threonine protein kinase	Protein phosphorylation
AALF020589	tRNA pseudouridine synthase	RNA modification	AALF019930^a^	Unspecified product	Lipid transport
AALF022027	Gdp mannose-4 2C6-dehydratase	GDP-mannose metabolic process	AALF022150	Histone H2B	Protein heterodimerization activity
AALF022345	Unspecified product	ATP binding	AALF022690	Unspecified product	NA
AALF022346^b^	Actin-1	Nucleotide binding	AALF024624	Histone H4	DNA-templated transcription initiation
AALF023299	atbf1	Regulation of DNA-templated transcription	AALF024650	Spectrin	Actin filament capping
AALF023780^b^	Unspecified product	Tetrahydrobiopterin biosynthetic process	AALF025146	Glycerol kinase	Glycerol-3-phosphate metabolic process
AALF026435	Histone H2B	Nucleosome assembly	AALF025156	Putative 40 s ribosomal protein s2	Translation
AALF027368^a,b^	Unspecified product	Lipid transport	AALF027368	Unspecified product	Lipid transport

Fo			Tap		
Accession no.	Name	GO (MF/BP)	Accession no.	Name	GO (MF/BP)
Four days pBM					
AALF000831	Unspecified product	Proteolysis	AALF000045	Unspecified product	Protein catabolic process
AALF001196^c^	C-Type Lectin (CTL)-mannose binding	Carbohydrate binding	AALF001196^c^	C-Type Lectin (CTL)-mannose binding	Carbohydrate binding
AALF002028^c^	Ran-binding protein	Intracellular transport	AALF001261	Glutathione transferase	Protein binding
AALF002713	Protein regulator of cytokinesis 1 prc1	Microtubule cytoskeleton organization	AALF002028^c^	Ran-binding protein	Intracellular transport
AALF003152	Heat shock cognate 70 isoform B	Protein folding	AALF002114	Cell division control protein	DNA replication initiation
AALF003990	Mannosyltransferase	Glycosyltransferase activity	AALF003247	Mannosyltransferase 1 2C	Protein O-linked glycosylation
AALF005662^c^	Lethal(2)essential for life protein 2 C l2efl	Metal ion binding	AALF003502	Kynurenine aminotransferase	Kynurenine metabolic process
AALF006045	Unspecified product	NA	AALF005085	ATP-binding cassette transporter	Transmembrane transport
AALF006185	Unspecified product	NA	AALF005662^c^	Lethal(2)essential for life protein 2 C l2efl	Metal ion binding
AALF007667^c^	Elongation factor 1-alpha	Translational elongation	AALF005708	Unspecified product	Cytoskeleton organization
AALF008063	Unspecified product	Protein binding	AALF007572	Unspecified product	Protein binding
AALF008379^c^	Unspecified product	Lipid transport	AALF007667^c^	Elongation factor 1-alpha	Translational elongation
AALF008766^c^	Vitellogenin-A1 precursor	Lipid transport	AALF007724	ATP synthase subunit beta	Proton motive force-driven ATP synthesis
AALF009209^c^	Serine/threonine kinase	Protein phosphorylation	AALF008130	Glycogen synthase	Glycogen biosynthetic process
AALF010133^c^	Elongation factor 1-alpha	Translational elongation	AALF008379^c^	Unspecified product	Lipid transport
AALF010343^c^	Unspecified product	Carbohydrate binding	AALF008766^c^	Vitellogenin-A1 precursor	Lipid transport
AALF010627	Unspecified product	Protein binding	AALF009209^c^	Serine/threonine_kinase	Protein phosphorylation
AALF011026^c^	Unspecified product	Nucleic acid binding	AALF009854	Unspecified product	Protein binding
AALF011409^c^	Unspecified product	Proteolysis	AALF010133^c^	Elongation factor 1-alpha	Translational elongation
AALF012426	Signal recognition particle receptor alpha subunit	Intracellular protein transport	AALF010304	Calcium-binding protein E63-1	Calcium ion binding
AALF012812	Calmin	Cellular component organization	AALF010343^c^	Unspecified product	Carbohydrate binding
AALF012831	Calcium transporting ATPase 2 (ATPase 2)	Calcium ion transmembrane transport	AALF011026^c^	Unspecified product	Nucleic acid binding
AALF016841^c^	Cathepsin b	Proteolysis	AALF011409^c^	Unspecified product	Proteolysis
AALF017232^c^	Cathepsin b	Proteolysis	AALF011822	Leucine-rich immune protein (Coil-less)	Protein binding
AALF017233^c^	Unspecified product	Proteolysis	AALF011930	Unspecified product	Nucleic acid binding
AALF017331	Amidophosphoribosyltransferase	Purine nucleotide biosynthetic process	AALF012374	GTP:AMP phosphotransferase	ITP metabolic process
AALF017662	Oviductin	Proteolysis	AALF013007	Protein tyrosine phosphatase 69d	Protein dephosphorylation
AALF018200	GTP-binding protein alpha subunit 2 C gna	Signal transduction	AALF013502	Translation initiation factor	Cellular metabolic process
AALF018275^c^	Unspecified product	Proteolysis	AALF016841^c^	Cathepsin b	Proteolysis
AALF018689	Unspecified product	Nitrogen compound metabolic process	AALF017233^c^	Unspecified product	Proteolysis
AALF018796	DNA polymerase v	Regulation of DNA transcription	AALF017772	Unspecified product	NA
AALF019930^c^	Unspecified product	Lipid transport	AALF018275^c^	Unspecified product	Proteolysis
AALF020554^c^	Actin	Nucleotide binding	AALF018477	Non-specific serine/threonine protein kinase	Protein phosphorylation
AALF020972	Lipase	Lipid metabolic process	AALF019930^c^	Unspecified product	Lipid transport
AALF021555	Unspecified product	ATP binding	AALF020554^c^	Actin	Nucleotide binding
AALF022346^c^	Actin-1	Nucleotide binding	AALF020589	tRNA pseudouridine synthase	RNA modification
AALF022559	Alcohol dehydrogenase	Obsolete oxidation–reduction process	AALF022027	Gdp mannose-4 2C6-dehydratase	GDP-mannose metabolic process
AALF022690^c^	Unspecified product	NA	AALF022345	Unspecified product	ATP binding
AALF023780^c^	Unspecified product	Tetrahydrobiopterin biosynthetic process	AALF022346^c^	Actin-1	Nucleotide binding
AALF024081^c^	Carboxypeptidase	Proteolysis	AALF022690^c^	Unspecified product	NA
AALF025260	Oviductin	Proteolysis	AALF023780^c^	Unspecified product	Tetrahydrobiopterin biosynthetic process
AALF025991	Histone-lysine n-methyltransferase	Histone lysine methylation	AALF024081^c^	Carboxypeptidase	Proteolysis
AALF026483	Unspecified product	Eukaryotic translation initiation	AALF025494	Glyceraldehyde-3-phosphate dehydrogenase	Glycolytic process
AALF026901	Sdk-P1	Anatomical structure morphogenesis	AALF026319	Tropomyosin invertebrate	NA
AALF027134	Unspecified product	Protein binding	AALF026435	Histone H2B	Nucleosome assembly
AALF027368^c^	Unspecified product	Lipid transport	AALF027368^c^	Unspecified product	Lipid transport

Proteins listed in table were identified by liquid chromatography–tandem mass spectrometry analysis

*BP* Biological process,* Fo* Foshan reference strain,* GO* Gene Ontology,* MF* molecular function,* NA* not available,* pBM* post blood meal, * Tap* Tapachula (Mexico) population

^a^Proteins found in common between Fo and Tap at 1 day pBM

^b^Proteins in common between Fo at 1 day pBM and Tap at 4 days pMB

^c^Proteins found in common between Fo and Tap at 4 days pBM

### Invasive mosquitoes efficiently allocate nutritional resources during oogenesis

**Fig. 3:**
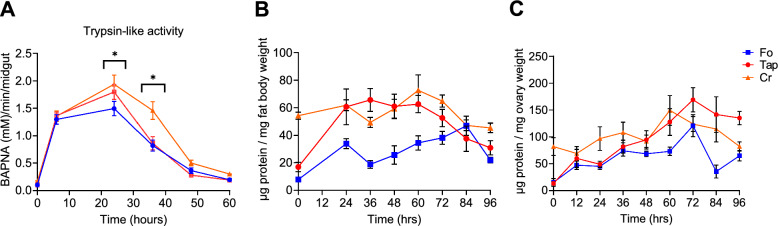
Oviposition-related metabolism. **A** Trypsin-like activity in female before blood meal (time 0) and at 10, 20, 30, 40, 50, and 60 h post blood meal (hpBM). Trypsin-like activity was detected using BAPNA, a chromogenic substrate. ** b**,** c** Protein content in the fat body (**B**) and ovaries (**C**) of females before the blood meal (time 0) and at 24, 36, 48, 60, 72, 84, and 96 hpBM. Asterisk indicates a significant difference at *P* < 0.05

To further probe the physiological basis of the observed delay in oviposition of invasive versus Fo females, we quantified trypsin-like enzyme activity after a blood meal, as a proxy for blood protein digestion [36]. We also measured and compared the accumulation and depletion of energy reserves in the fat body and their absorption in the ovaries (Fig. 3) because after acquisition and digestion of a blood meal, yolk precursor proteins are produced in the fat body, secreted into the hemolymph and transported to oocytes for egg production [37].

Mosquitoes aged between 3 and 5 days were weighed and then provided with a blood meal; after mosquitoes had flown to a resting spot, we weighed them again to avoid having the quantification of blood intake biased by diuresis (Additional file 3: Figure S2). The protease activity of trypsin-like enzymes showed similar trends in Fo, Tap and Cr females (Fig. 3a). Protease activity was detected from 6 to 60 hpBM, with a peak at 24 hpBM in all tested populations, but a significantly higher trypsin-like activity was found in invasive versus Fo mosquitoes at 24 hpBM (Fig. 3a). These results suggest that invasive mosquitoes process a blood meal more efficiently than those of the Fo reference population. To confirm this result, we tracked the mobilization of proteins from the fat body to ovaries post blood meal at 12-h intervals for up to 4 days pBM (Fig. 3 b, c). Similar patterns were observed in Tap and Cr females, with protein accumulation in the fat body gradually increasing up to 60–72 hpBM, then gradually decreasing, and reaching its minimum level at 96 hpBM. In contrast, protein content in the fat body of Fo females decreased at 36 hpBM and then gradually increased, reaching its maximum level at 84 hpBM, the once again declining at 96 hpBM (Fig. 3b). Protein mobilization from the fat body to the ovary showed a similar pattern across populations, with a gradual increase and a peak at 60 hpBM in Cr mosquitoes and at 72 hpBM in Fo and Tap mosquitoes, followed by a marked decrease only in Fo mosquitoes at 84 hpBM (Fig. 3c); these results are in agreement with an earlier start of oviposition (Fig. 1d). Monitoring of lipids revealed that there was a gradual accumulation of lipids in the ovaries of Tap and Fo mosquitoes, followed by a decrease at 60 or 72 hpBM in Fo or Tap mosquitoes, respectively (Additional file 4: Figure S3).

### Variation in levels of heterosis

**Fig. 4:**
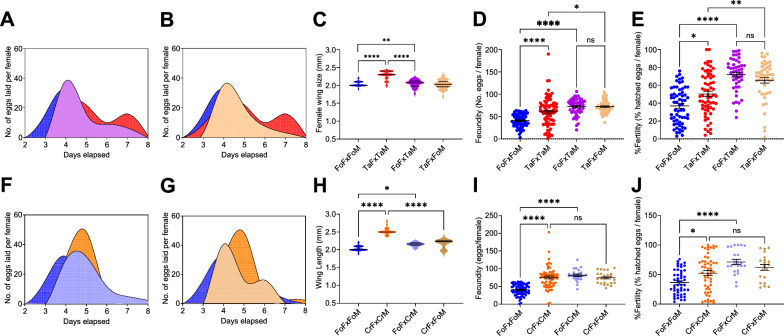
Varying levels of heterosis in crosses with invasive mosquitoes. **A** Oviposition patterns of F1 progenies of crosses between females of the Foshan reference strain (Fo) and males of the Tapachula population (Tap), in comparison with that of the parental populations. **B** Oviposition patterns of F1 progenies of crosses between Fo males and Tap females in comparison to that of the parental populations.** C–E** Wing size (**C**) fecundity (**D**) and fertility (**E**) of F1 progeny of crosses between Fo females and Tap males or Fo males and Tap females, in comparison to that of the parental populations. **F** Oviposition patterns of F1 progenies of crosses between Fo females with Crema (Cr) males with respect to that of parental populations. **G** Oviposition patterns of F1 progenies of crosses between Cr males with Fo females in comparison to that of parental populations. **H–J** Wing size (**H**), fecundity (**I**) and fertility (**J**) of F1 progeny of crosses between Fo females and Cr males or Fo males and Cr females in comparison with that of parental populations. Asterisks indicate a significant difference at *P < 0.05, **P < 0.01, ***P < 0.001 and ****P < 0.0001; ns, not significant

To test for heterosis, we established reciprocal crosses between Tap and Fo and between Cr and Fo mosquitoes. We measured the wing length, fecundity and fertility of F1 females of all reciprocal crosses and monitored their oviposition patterns, and compared these to these traits of the respective parents (Fig. 4). In the matings between Tap and Fo mosquitoes, the F1 progeny showed higher fecundity and fertility than both parental strains, supporting heterosis (Fig. 4d, e; Additional File 2: Table S5). We also observed that the percentage of sterile females was significantly lower in the F1 progeny of crosses between Fo females and Tap males in comparison with the crosses of parental Fo, but not parental Tap mosquitoes (*F* = 6.538, *P *= 0.0152) (Additional file 5: Figure S4). The same trend was observed for egg viability (Additional file 5: Figure S4), suggesting a contribution of Tap males to modulating the reproductive output of Fo females. In the matings between the Cr and Fo mosquitoes, the F1 progeny showed a higher reproductive output than the parental Fo, but not significantly different than that of the invasive Cr mosquitoes (Fig. 4h–l). In both crosses, the wing length and the oviposition patterns of the F1 progeny showed intermediate phenotypes compared to those of the parental populations, suggesting Mendelian inheritance of quantitative traits (Fig. 4).

## Discussion

Reproduction is an essential process to ensure the survival of a species and a key determinant of the success of a species invasion because it facilitates the ability of a species to establish itself in a new area. In the present study, we have shifted the focus from species to populations, and we show differences in the reproductive capacity across *Ae. albopictus* populations, which can be associated with their history. The limited number of populations we studied and particularly the use of a single long-established laboratory colony from the native range constrain our ability to generalize our results. Observed phenotypic differences likely reflect the contribution of both population differences and laboratory adaptation. However, the observed temporal stability in fitness traits across our populations (Additional file 1: Figure S1; Fig. 4) [22], support the conclusion that their genetic background contributes to observed differences. All our invasive populations showed a consistent pattern, namely they exhibit higher reproductive output compared to mosquitoes from both the long laboratory adapted Foshan reference population and the LaR population. These repeated associations prompted us to investigate their underlying biological mechanisms. We observed that the higher reproductive output of mosquitoes from invasive populations has physiological basis: these mosquitoes optimized their nutrient investment during oogenesis, resulting in increased egg production associated with a delay in oogenesis. In *Aedes* spp. mosquitoes, the acquisition of a blood meal and its digestion stimulate vitellogenesis and change the metabolic activity of the fat body, resulting in the production of yolk precursor proteins, the secretion of yolk proteins and lipids, and the deposition of yolk proteins and lipids in ovaries [36, 37, 41–43]. Oviposition-related metabolism also promotes morphological and physiological changes in ovaries during oogenesis [37]. *Aedes* spp. females emerge with two ovaries, each divided into 50–60 ovarioles containing an oocyte, nurse cells and surrounding follicle cells forming the primary egg chamber [37]. During vitellogenesis, developing oocytes grow synchronously through the acquisition of proteins, lipids and carbohydrates to form mature eggs [37]. The oocyte of the secondary egg chamber will develop into a mature egg after a secondary blood meal [37]. Egg production is cyclical. A round of ovarian development from blood intake to egg laying is called a gonotrophic cycle. We observed that the fat body and ovaries of the invasive Tap and Cr mosquitoes had a higher protein content than those of their Fo counterparts, suggesting optimized blood meal digestion to allocate resources for egg production. In the ovaries of Tap females, lipid increase was coordinated with protein accumulation, while lipid flux and protein deposition into the developing ovaries did not follow a similar trend in Fo females. Lipids appeared later in the ovaries of Tap females than in those of Fo females, resulting in a delay in oogenesis and egg deposition. However, the more efficient acquisition of nutrients allowed females from invasive Tap and Cr populations to sustain egg production longer than Fo, resulting in higher fecundity and fertility. The observed delay in the oogenesis of Tap and Cr mosquitoes indicates that maximization of allotment in the first clutch of eggs is associated with substantial benefits. Indeed, life history theory predicts that early reproduction is more likely to be successful, and therefore more valuable, than later reproduction because the chance of mortality increases with age [44]. We also verified that the delay in oviposition of Tap females does not extend to the second GC, probably because sufficient energetic resources are available in the fat body. We cannot exclude that the shift of nutrients from the blood meal to reproduction may result in a fitness cost in terms of longevity.

*Aedes albopictus* moved globally in less than 60 years, with recurrent incursions favouring genetic admixture [11]. This invasion dynamics and our results showing varying degree of heterosis in crosses involving invasive mosquitoes support the hypothesis that the observed differences in the reproductive capacity across populations are adaptive. The hypothesis that *Ae. albopictus* can undergo rapid adaptation is supported the results of a study on the distribution of mutations predictive of pyrethroid resistance, which have appeared rapidly and independently across different populations [45]. Moreover, there are frequent examples of invasive species adapting quickly to new environments. A well-studied example is the rapid evolution of *Drosophila suboscura* in the New World, which was demonstrated by the detection of rapidly evolving concordant patterns of chromosomal inversions across latitudinal clines in three different continents [46]. In addition to being facilitated by intraspecific hybridization in the introduced range, which can result in heterosis and lead to novel genotypes, rapid adaptation in *Ae. albopictus* reproductive capacity could be induced by severe changes in the selection regime imposed by new environments [47]. As an alternative explanation for the hypothesis of rapid adaptation, *Ae. albopictus* invasive populations may have a high reproductive capacity because they derive from regions prone to ecological disturbance. It has been hypothesized that fluctuating environments might favour organismal flexibility or the selection for evolvability by accumulation and maintenance of genetic variation [48]. In fact, recent population genomic studies showed that the invasive and native populations of *Ae. albopictus* retain similar levels of genomic diversity and that invasive populations can become sources of new founder populations promoting further invasions [12, 35].

## Conclusions

The observed differences in the reproductive capacity of *Ae. albopictus* populations in this study are underpinned by physiological adjustments and hybrid vigour. Such traits can be associated with a successful invasion by enhancing the reproductive output and persistence in novel environments. More broadly, our findings emphasize that the success of biological invasions may be influenced by intraspecific variation across diverse populations.

## Supplementary Information

Additional file 1.

Additional file 2.

Additional file 3.

Additional file 4.

Additional file 5.

## Data Availability

Whole Genome Sequencing data that support the findings of this study are openly available in NIH SRA BioProject number PRJNA1080321. Scripts and our custom genome wide GO annotations for* Aedes albopictus*, based on the reference genome AalbF2 from VectorBase version 61, have been deposited in the publicly accessible repository https://github.com/naborlozada/Khorramnejad_et_al_2024.

## Abbreviations

Cr: Crema strain
Fo: Foshan strain
GC: Gonadotrophic cycle
LaR: La Reunion Island strain
pBM: Post blood meal
Pv: Pavia strain
SF: Sugar-fed
Tap: Tapachula strain

## References

[1] Elton CS. The ecology of invasions by animals and plants. New York: Springer; 1958.

[2] Pysek P, Richardson DM. Traits associated with invasiveness in alien plants: where do we stand? In: Nentwig W, editor. Biological invasions. Ecological studies 193. Heidelberg/Berlin/New York: Springer; 2007. p. 97–125.

[3] Capellini I, Baker J, Allen WL, et al. The role of life history traits in mammalian invasion success. Ecol Lett. 2015;18:1099–107. 26293900 10.1111/ele.12493 PMC4989474

[4] Poidatz J, Bressac C, Bonnard O, et al. Comparison of reproductive traits of foundresses in a native and an invasive hornet in Europe. J Insect Physiol. 2018;109:93–9. 30006107 10.1016/j.jinsphys.2018.07.004

[5] Mathakutha R, Steyn C, le Roux P, et al. Invasive species differ in key functional traits from native and non-invasive alien plant species. J Veg Sci. 2019;30:994–1006.

[6] Rejmanek M, Richardson DM. What attributes make some plant species more invasive? Ecology. 1996;77:1655–61.

[7] Mondor EB, Tremblay MN, Messing RH. Morphological and ecological traits promoting aphid colonization of the Hawaiian Islands. Biol Invasions. 2007;9:87–100.

[8] Laugier GJM, Le Moguédec G, Tayeh A, et al. Increase in male reproductive success and female reproductive investment in invasive populations of the harlequin ladybird *Harmonia axyridis*. PLoS ONE. 2013;8:1–10. 10.1371/journal.pone.0077083 PMC3799855 24204741

[9] Wessels FJ, Kristal R, Netter F, et al. Does it pay to delay? Flesh flies show adaptive plasticity in reproductive timing. Oecologia. 2011;165:311–20. 20953961 10.1007/s00442-010-1805-z PMC3739455

[10] Delatte H, Bagny L, Brengue C, et al. The invaders: phylogeography of dengue and chikungunya viruses *Aedes* vectors, on the South West islands of the Indian Ocean. Infect Genet Evol. 2011;11:1769–81. 21827872 10.1016/j.meegid.2011.07.016

[11] Bonizzoni M, Gasperi G, Chen X, et al. The invasive mosquito species *Aedes albopictus:* current knowledge and future perspectives. Trends Parasitol. 2013;29:460–8. 23916878 10.1016/j.pt.2013.07.003 PMC3777778

[12] Manni M, Guglielmino CR, Scolari F, et al. Genetic evidence for a worldwide chaotic dispersion pattern of the arbovirus vector, *Aedes albopictus*. PLoS Negl Trop Dis. 2017;11:e0005332. 10.1371/journal.pntd.0005332. 10.1371/journal.pntd.0005332 PMC5300280 28135274

[13] Pichler V, Kotsakiozi P, Caputo B, et al. Complex interplay of evolutionary forces shaping population genomic structure of invasive *Aedes albopictus* in southern Europe. PLoS Negl Trop Dis. 2019;13:1–24. 10.1371/journal.pntd.0007554 PMC6705758 31437154

[14] Vavassori L, Honnen AC, Saarman N, et al. Multiple introductions and overwintering shape the progressive invasion of *Aedes albopictus* beyond the Alps. Ecol Evol. 2022;12:1–19. 10.1002/ece3.9138 PMC9313497 35903757

[15] Li Y, Stift M, van Kleunen M. Admixture increases performance of an invasive plant beyond first-generation heterosis. J Ecol. 2018;106:1595–606.

[16] van Kleunen M, Röckle M, Stift M. Admixture between native and invasive populations may increase invasiveness of *Mimulus guttatus*. Proc R Soc Lond B Biol Sci. 2015;282(1815):20151487. 10.1098/rspb.2015.1487. 10.1098/rspb.2015.1487 PMC4614753 26354937

[17] Hahn MA, Rieseberg LH. Genetic admixture and heterosis may enhance the invasiveness of common ragweed. Evol Appl. 2017;10:241–50. 28250809 10.1111/eva.12445 PMC5322403

[18] Fournier D, Aron S. Hybridization and invasiveness in social insects—the good, the bad and the hybrid. Curr Opin Insect Sci. 2021;46:1–9. 33484875 10.1016/j.cois.2020.12.004

[19] Ekechukwu NE, Baeshen R, Traorè SF, et al. Heterosis increases fertility, fecundity, and survival of laboratory-produced F1 hybrid males of the malaria mosquito *Anopheles coluzzii*. G3 (Bethesda). 2015;5:2693–709. 26497140 10.1534/g3.115.021436 PMC4683642

[20] Lan L, Nègre N. Heterosis effect for larval performance of fall armyworm interstrain hybrids. Insect Sci. 2024;31:1296–312. 37969057 10.1111/1744-7917.13295

[21] Palatini U, Masri RA, Cosme LV, et al. Improved reference genome of the arboviral vector *Aedes albopictus*. Genome Biol. 2020;21:1–29. 10.1186/s13059-020-02141-w PMC7448346 32847630

[22] Carlassara M, Khorramnejad A, Oker H, et al. Population-specific responses to developmental temperature in the arboviral vector *Aedes albopictus*: Implications for climate change. Glob Chang Biol. 2024;30:1–22. 10.1111/gcb.17226 38454541

[23] Ramachandran KM, Tsokos CP. Mathematical statistics with applications. London: Academic Press; 2020.

[24] Gulia-Nuss M, Eum JH, Strand MR, et al. Ovary ecdysteroidogenic hormone activates egg maturation in the mosquito *Georgecraigius atropalpus* after adult eclosion or a blood meal. J Exp Biol. 2012;215:3758–67. 22811249 10.1242/jeb.074617 PMC3470065

[25] Geiser DL, Li W, Pham DQD, et al. Shotgun and TMT-labeled proteomic analysis of the ovarian proteins of an insect vector, *Aedes aegypti* (Diptera: Culicidae). J Insect Sci. 2022;22:7. 10.1093/jisesa/ieac018. 10.1093/jisesa/ieac018 PMC8932505 35303100

[26] Noble JE, Bailey MJA. Quantitation of protein. Methods Enzymol. 2009;463:73–95. 19892168 10.1016/S0076-6879(09)63008-1

[27] Lozada-Chávez AN, Lozada-Chávez I, Alfano N, et al. Adaptive genomic signatures of globally invasive populations of the yellow fever mosquito *Aedes aegypti*. Nat Ecol Evol. 2025;9:652–71. 40155778 10.1038/s41559-025-02643-5 PMC11976285

[28] Wu T, Hu E, Xu S, et al. ClusterProfiler 4.0: a universal enrichment tool for interpreting omics data. The Innov. 2021;2:100141. 10.1016/j.xinn.2021.100141 PMC8454663 34557778

[29] Andrews S. FastQC: A quality control tool for high throughput sequence data. 2010. https://www.bioinformatics.babraham.ac.uk/projects/fastqc/. Accessed 1 Sep 2023.

[30] Chen S. Ultrafast one-pass FASTQ data processing, quality control, and deduplication using fastp. iMeta. 2023;2:e107. 38868435 10.1002/imt2.107 PMC10989850

[31] Giraldo-Calderón GI. VectorBase.org updates: bioinformatic resources for invertebrate vectors of human pathogens and related organisms. Curr Opin Insect Sci. 2022;50:100860. 34864248 10.1016/j.cois.2021.11.008 PMC9133010

[32] Vasimuddin M, Misra S, Li H, et al. Efficient architecture-aware acceleration of BWA-MEM for Multiscore Systems. In: International Parallel and Distributted Processing Symposium. 20–24 May 2019, Rio de Janeiro. p. 314–24.

[33] Czech L, Spence JP, Expósito-Alonso M. greendalf: population genetic statistics for the next generation of pool sequencing. arXiv. 2024. https://arxiv.org/abs/2306.11622. 10.1093/bioinformatics/btae508 PMC11357794 39185959

[34] Kofler R, Pandey RV, Schlotterer C. PoPoolation2: identifying differentiation between populations using sequencing of pooled DNA samples (Pool-Seq). Bioinformatics. 2011;27:3435–6. 22025480 10.1093/bioinformatics/btr589 PMC3232374

[35] Gloria-Soria A, Shragai T, Ciota A, et al. Population genetics of an invasive mosquito vector, *Aedes albopictus* in the Northeastern USA. NeoBiota. 2022;78:99–127. 37408738 10.3897/neobiota.78.84986 PMC10321554

[36] Clements AN. The biology of mosquitoes volume 1: development, nutrition and reproduction. Wallingford: CAB International; 1992.

[37] Valzania L, Mattee MT, Strand MR, et al. Blood feeding activates the vitellogenic stage of oogenesis in the mosquito *Aedes aegypti* through inhibition of glycogen synthase kinase 3 by the insulin and TOR pathways. Dev Biol. 2019;454:85–95. 31153832 10.1016/j.ydbio.2019.05.011 PMC6717557

[38] Moreau CA, Bhargav SP, Kumar H, et al. A unique profilin-actin interface is important for malaria parasite motility. PLoS Pathog. 2017;13:e1006412. 10.1371/journal.ppat.1006412. 10.1371/journal.ppat.1006412 PMC5464670 28552953

[39] Dhadialla TS, Raikhel AS. Biosynthesis of mosquito vitellogenin. J Biol Chem. 1990;265:9924–33. 2351682

[40] do Nascimento Neto JF, da Mota AJ, Roque RA, et al. Analysis of the transcription of genes encoding heat shock proteins (hsp) in *Aedes aegypt*i Linnaeus, 1762 (Diptera: Culicidae), maintained under climatic conditions provided by the IPCC (Intergovernmental Panel On Climate Change) for the year 2100. Infect Genet Evol. 2020;86:1–8. 10.1016/j.meegid.2020.104626 33166684

[41] Gondim KC, Atella GC, Pontes EG, et al. Lipid metabolism in insect disease vectors. Insect Biochem Mol Biol. 2018;101:108–23. 30171905 10.1016/j.ibmb.2018.08.005

[42] Price DP, Nagarajan V, Churbanov A, et al. The fat body transcriptomes of the yellow fever mosquito *Aedes aegypti,* pre- and post- blood meal. PLoS ONE. 2011;6:e22573. 10.1371/journal.pone.0022573. 10.1371/journal.pone.0022573 PMC3144915 21818341

[43] Santiago PB, De Araújo CN, Motta FN, et al. Proteases of haematophagous arthropod vectors are involved in blood-feeding, yolk formation and immunity—a review. Parasit Vectors. 2017;10:1–20. 28193252 10.1186/s13071-017-2005-z PMC5307778

[44] Moreno Klemming, J. Reproductive success. In: Choe JC, editor. Encyclopedia of animal behavior. Cambridge: Academic Press; 2019. p. 94–100.

[45] Tancredi A, Papandrea D, Marconcini M, et al. Tracing temporal and geographic distribution of resistance to pyrethroids in the arboviral vector *Aedes albopictus*. PLoS Negl Trop Dis. 2020;14:1–17. 10.1371/journal.pntd.0008350 PMC7332087 32569337

[46] Huey RB, Gilchrist GW, Hendry AP. Using invasive species to study evolution: case studies with *Drosophila* and salmon. In: Species invasions: insights into ecology, evolution, and biogeography. Sunderland: Sinauer Associates; 2005. p. 39–164.

[47] Sakai AK, Allendorf FW, Holt JS, et al. The population biology of invasive species. Annu Rev Ecol Evol Syst. 2001;32:305–32.

[48] Lee CE, Gelembiuk GW. Evolutionary origins of invasive populations. Evol Appl. 2008;1:427–48. 25567726 10.1111/j.1752-4571.2008.00039.x PMC3352381

