# Supplementary figures and images for: Population differences in reproductive resource allocation and heterosis in the invasive vector Aedes albopictus
Source: Parasit Vectors. 2026 Jan 13;19:77. doi: 10.1186/s13071-025-07235-7 (PMC12888394; doi:10.1186/s13071-025-07235-7)

**A**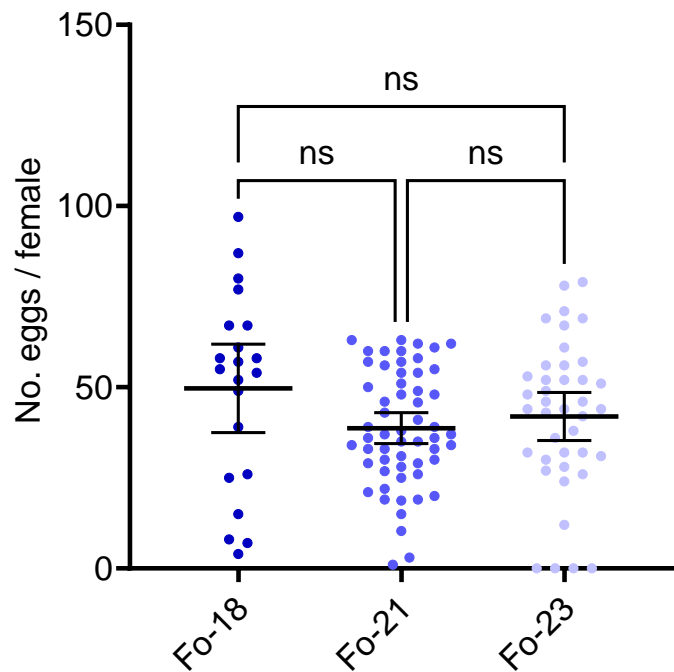**B**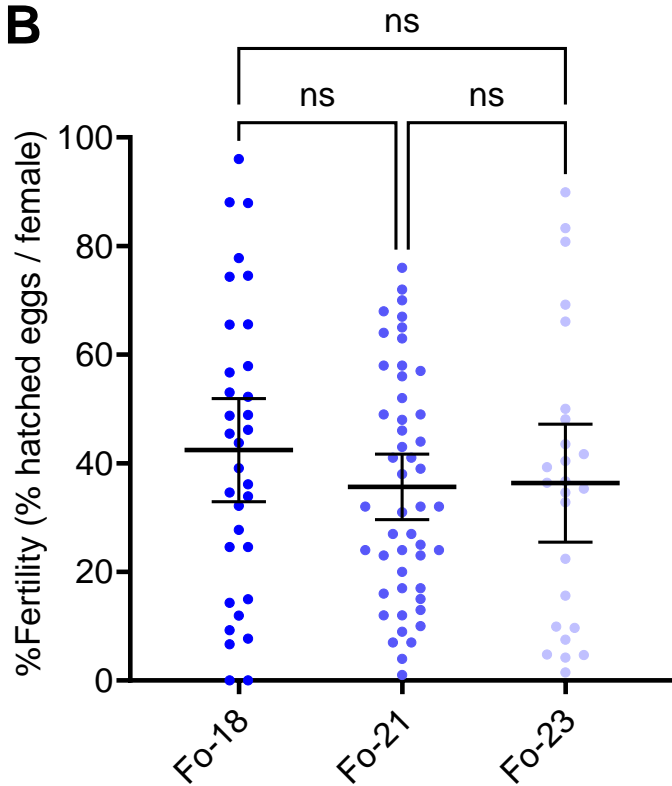

Supplement: Supplementary file 1 — Additional file 1. [file 13071_2025_7235_MOESM1_ESM.pdf]

# 1st GC

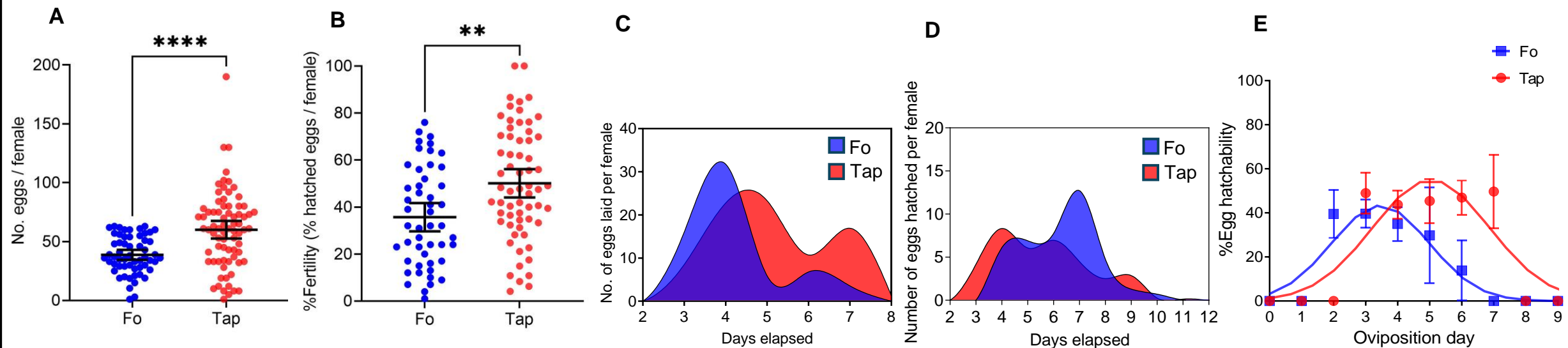

# 2nd GC

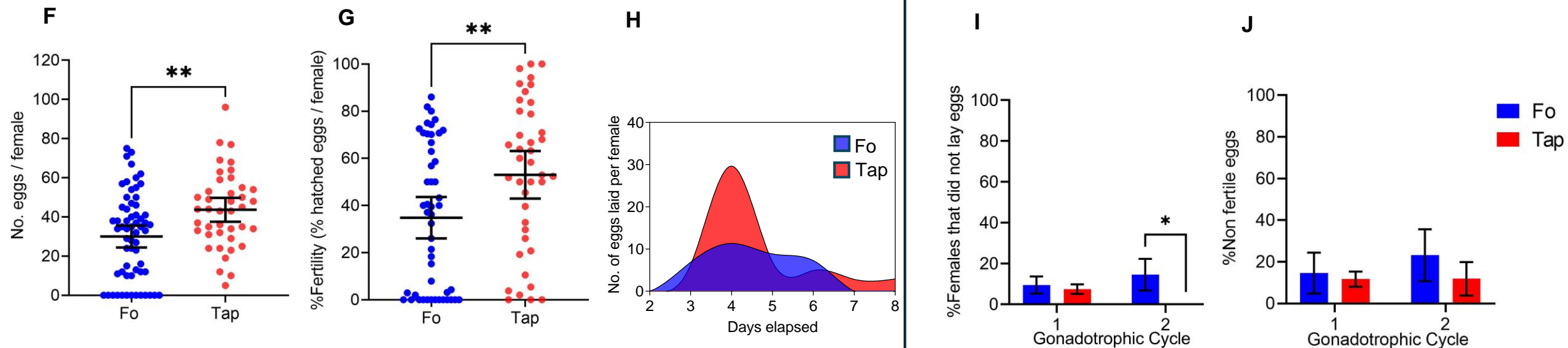

Supplement: Supplementary file 2 — Additional file 2. [file 13071_2025_7235_MOESM2_ESM.pdf]

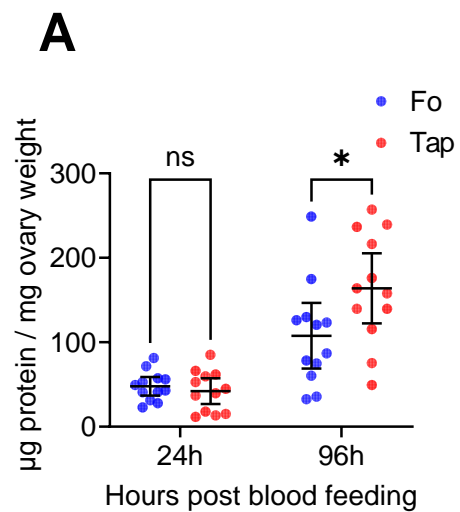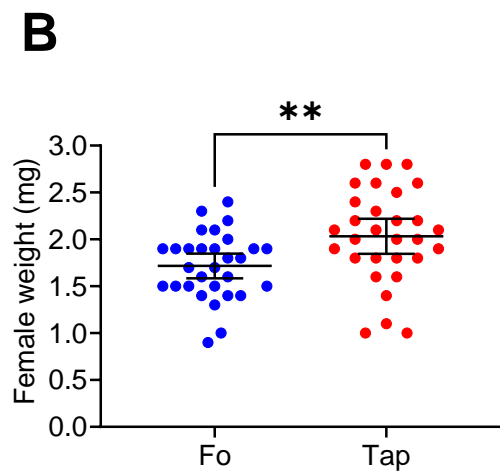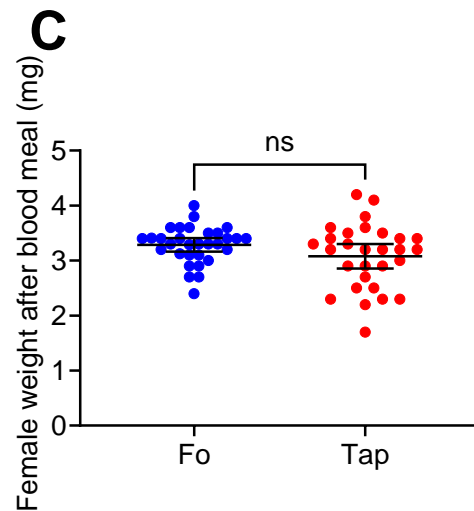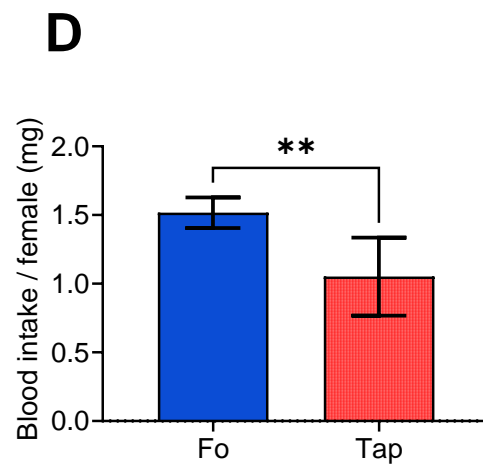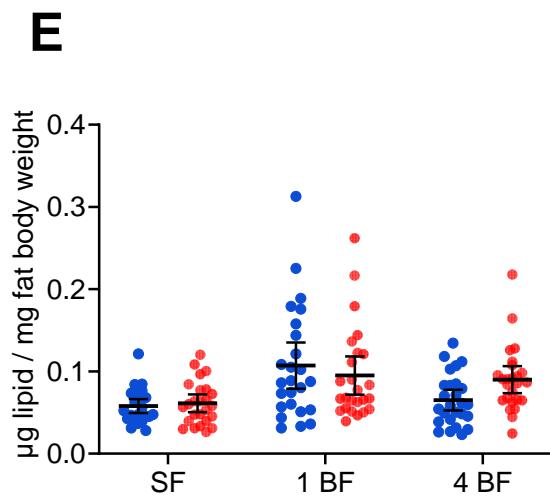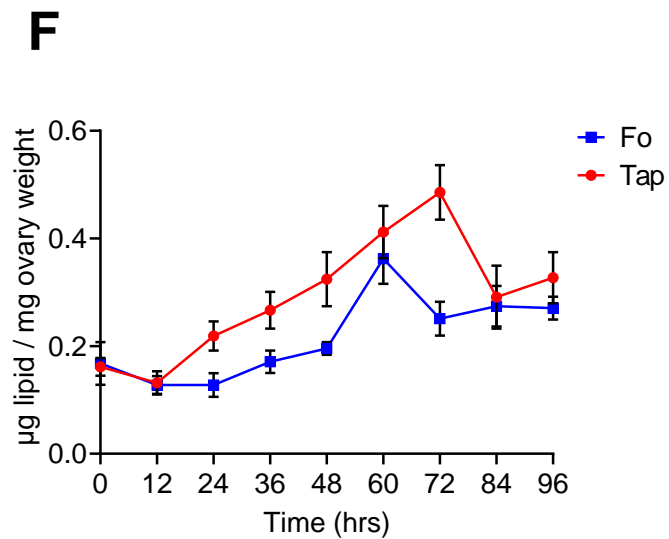

Supplement: Supplementary file 3 — Additional file 3. [file 13071_2025_7235_MOESM3_ESM.pdf]

**A**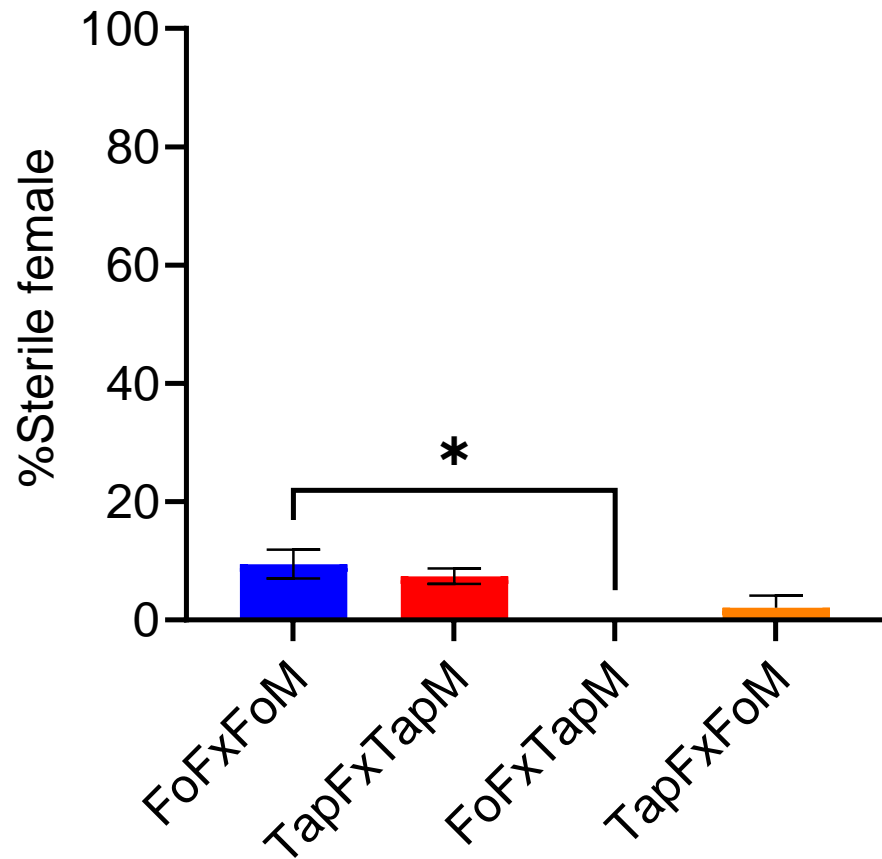**B**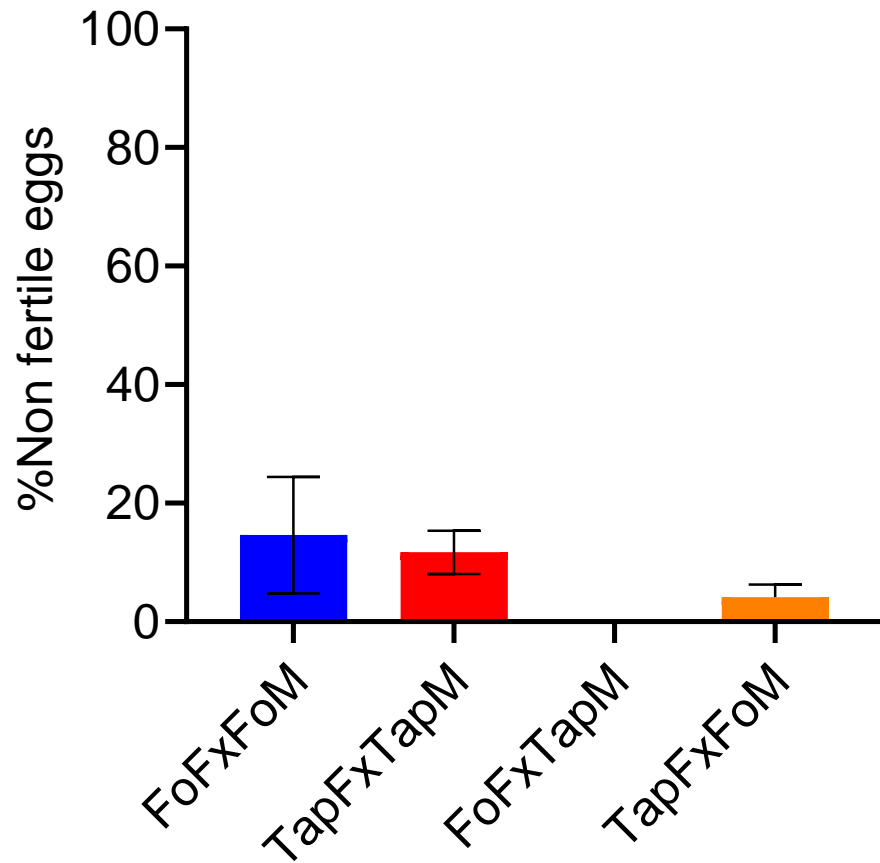

Supplement: Supplementary file 4 — Additional file 4. [file 13071_2025_7235_MOESM4_ESM.pdf]
